# Effects of Kinesio taping on lower limb biomechanical characteristics during the cutting maneuver in athletes after anterior cruciate ligament reconstruction

**DOI:** 10.1371/journal.pone.0299216

**Published:** 2024-03-07

**Authors:** Sizhuo Zhang, Ling Wang, Xiaoqian Liu, Guanglan Wang, Peng Chen

**Affiliations:** 1 Wuhan Business University, Wuhan, Hubei Province, China; 2 Key Laboratory of Sports Engineering of General Administration of Sport of China, Wuhan Sports University, Wuhan, Hubei Province, China; 3 School of Sports Medicine, Wuhan Sports University, Wuhan, Hubei Province, China; 4 School of Exercise and Health, Shanghai University of Sport, Shanghai, China; King Khalid University, SAUDI ARABIA

## Abstract

**Purpose:**

To determine the effects of Kinesio taping (KT) on the biomechanical characteristics of the lower limbs during the 90° cutting maneuver in anterior cruciate ligament (ACL) reconstruction (ACLR) athletes.

**Method:**

Eighteen ACLR athletes were recruited and subjected randomly to three taping conditions, KT, placebo taping (PT), and no taping (NT), followed by a 90° cutting test. A nine-camera infrared high-speed motion capture system (Vicon, T40, 200 Hz) was used to record the kinematic parameters of the lower limbs during the cutting maneuver, and a three-dimensional dynamometer (Kistler, 1000 Hz) was used to record the kinetic parameters of the lower limbs. A one-way repeated measures analysis of variance was conducted to compare the differences in the lower limb kinematic and kinetic characteristics of ACLR athletes subjected to these interventions.

**Results:**

During the landing phase, the knee valgus angle reduced significantly with KT than with NT (95% confidence interval = −1.399 to −0.154; *P* = 0.025), whereas no significant difference was observed between PT and NT (95% confidence interval = −1.251 to 0.217; *P* = 0.236). No significant differences were observed in the other kinematic variables among the three taping conditions (*P* > 0.05). During the landing phase, no significant differences in the kinetic variables were observed among the three taping conditions (*P* > 0.05).

**Conclusions:**

Although KT does not improve the kinetic variables of athletes after ACLR during the 90° cutting maneuver, it reduces the knee valgus angle, which could reduce the risk of secondary ACL injury.

## 1. Introduction

Anterior cruciate ligament (ACL) rupture is a devastating knee injury that most commonly occurs whilst playing sports [1]. More than 250,000 people suffer from ACL injury each year [2]. Given the severe consequences, most scholars advocate the use of ACL reconstruction (ACLR) surgery to restore knee stability and allow patients to resume sports. However, despite significant advances in surgical techniques, most patients have persistent long-term deficits in lower limb muscle strength [3, 4] and neuromuscular activation [5] as well as altered lower limb biomechanics during athletic tasks [6, 7]. Additionally, almost 20–25% of postoperative athletes develop secondary ACL injuries [8, 9]. Compared with primary ACLR surgery, the failure rate of ACL revision surgery is significantly higher and the postoperative knee function recovery is worse [10]. Reducing the risk of secondary ACL injury and the heavy medical costs caused by ACL revision has become a challenging problem in sports medicine.

Meeting certain health criteria before resuming sports can reduce the risk of secondary injury [11, 12]. Current studies have recommended adequate quadriceps strength and single-leg hop distance limb symmetry index >90% as the criteria for resuming sports after ACLR [13, 14]. Despite achieving quadriceps strength and single-leg hop distance symmetry, athletes after ACLR continue to have abnormal lower limb biomechanics, suggestive of a high risk of secondary injury. Setting very stringent criteria for resuming sports may reduce secondary injury risk. However, because only 11% of ACLR patients fulfill the criteria to resume sports 9 months after ACLR [15], making the criteria more stringent is unlikely to solve the problem, and optimizing prevention strategies after resuming sports seems more logical.

Smaller knee flexion angles [16], larger knee valgus angles and moments [17], and higher ground reaction forces [18] during the landing phase have been identified as important factors for the risk of secondary injury in athletes after ACLR. From the perspective of injury mechanisms, prevention strategies should focus on increasing the knee flexion angle and decreasing the knee valgus angle during the landing phase. Recently, Kinesio taping (KT) has been proposed to prevent knee injuries associated with biomechanical deficits [19]. KT can decrease the knee valgus angle during the landing phase in healthy athletes [19]. Moreover, Choi et al. [20] showed that KT could enhance athletes’ quadriceps strength. Higher quadriceps strength contributes to a higher knee flexion angle during the landing phase and a lower ground reaction force [21]. These studies indicate that KT may help reduce the knee valgus angle and ground reaction force, thereby reducing the risk of secondary injuries in athletes after ACLR. However, no studies have examined the effects of KT on the biomechanics of lower limbs in athletes after ACLR.

Therefore, this study aimed to determine the effects of KT on the biomechanical characteristics of the lower limbs during the 90° cutting maneuver in ACLR athletes. We hypothesized that the KT optimizes the lower limb biomechanical characteristics of ACLR athletes.

## 2. Methods

### 2.1 Participants

A priori power analysis (G*power 3.1.2) was performed to determine the appropriate sample size for the study. The calculations showed that a sample size of 16 participants was required to achieve a power of 0.90 and an effect size of 0.4 at an alpha level of 0.05, using one-way repeated measures analysis of variance (ANOVA). Therefore, 18 ACLR athletes were recruited, and their general information is summarized in **Table 1**. The recruitment period started in June 2023 and ended in October 2023. **Table 2** shows the inclusion and exclusion criteria. All participants were informed about the test process, and they provided signed informed consent forms before data collection. This study was approved by the Medical Ethics Committee of Wuhan Sports University (Approval No. 2023089).

**Table 1:** General patient information.

Sex (female/male), n	Age, years	Height, cm	Body mass, kg	Injured side (left/right), n	Tegner score pre-injury	Time since surgery, months
8/10	21.8±3.3	174.5±9.6	65.6±10.8	5/13	8.3±0.7	16.8±5.4

**Table 2:** Inclusion and exclusion criteria.

Inclusion criteria	Exclusion criteria
(1) Complete unilateral anterior cruciate ligament injury treated with an autologous ipsilateral bone–patellar tendon–bone or hamstring tendon graft (semitendinosus and/or gracilis tendon)	(1) Concomitant Grade III knee ligament injury, full-thickness articular cartilage lesion, history of other lower limb surgery (in either limb), back pain, or lower limb injury in the previous 3 months
(2) Postoperative period ≥9 months	(2) Unable to complete the tasks
(3) Cleared to resume all high-level athletic activities by their surgeon and treating rehabilitation specialist and intended to return to cutting and pivoting sports regularly (≥50 hours/year)	(3) Missing motion-analysis data during hoping
(4) Single-leg horizontal hop distance limb symmetry index >90% in the single-leg horizontal hop test	
(5) Tegner score ≥7 pre-injury	

### 2.2 Taping procedure

The taping area was cleared of hair and wiped with 70% alcohol before taping. The affected side of each participant was subjected randomly to the following three taping conditions: KT, placebo taping (PT), and no taping (NT). Subsequent experimental data collection was performed for the three taping interventions.

**Fig 1:**
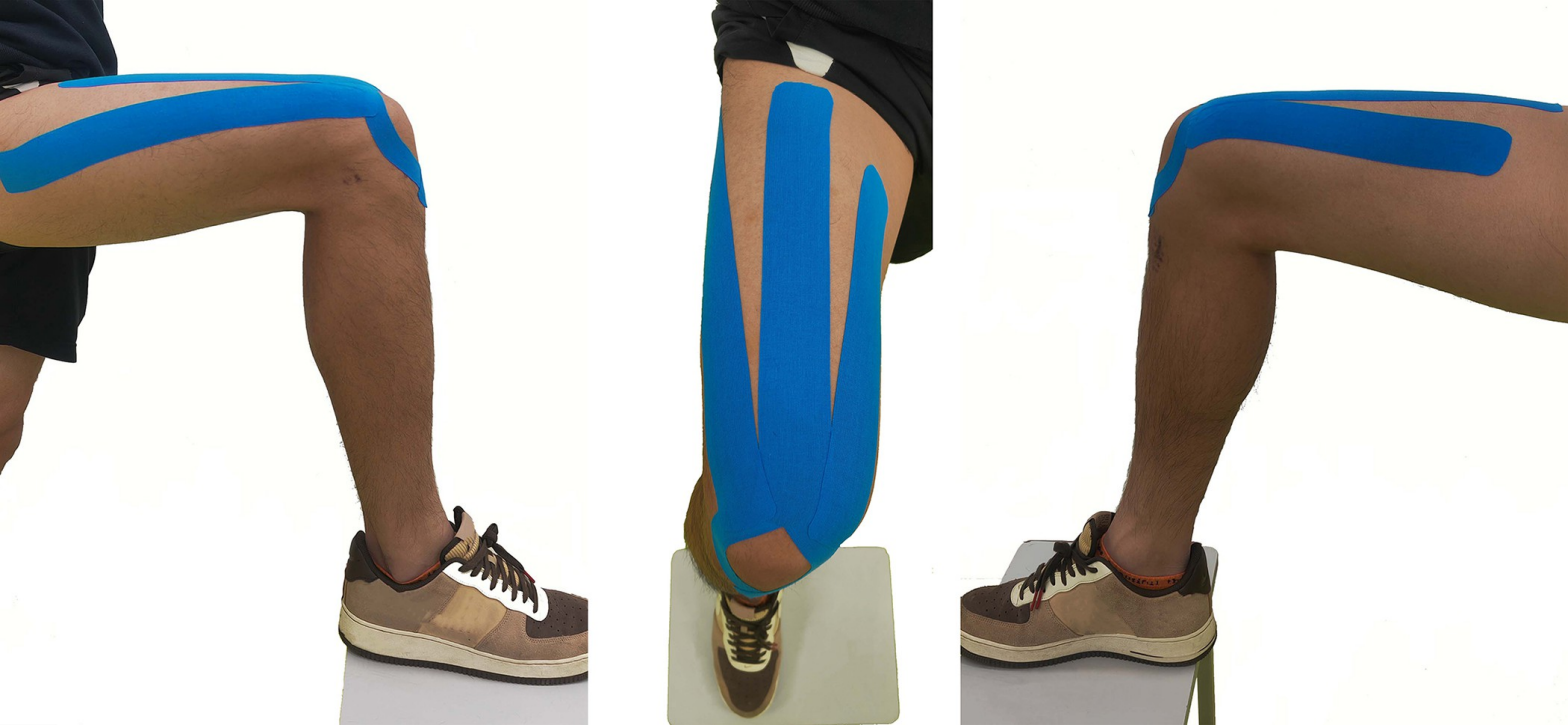
Kinesio taping applications used in the study.

KT (50 mm × 5 m) was applied on the rectus femoris, vastus lateralis, and vastus medialis muscles with a tension of 50% **(Fig 1)**. The formula for the taping cutting length calculation was as follows: Taping cutting length = ([actual length − 8 cm]/1.5 + 8 cm) × 1.1 [22, 23]. The KT application was performed with the participants standing on one foot, with the hip of the affected side at 0° and the knee flexed at 90°. For the rectus femoris, KT was applied from 10 cm below the anterior superior iliac spine to the tibial tuberosity and split in the form of “Y” above the patella. For the vastus lateralis muscle, KT was applied from the greater trochanter of the femur to the lateral edge of the patella. For the vastus medialis muscle, KT was applied from the middle third of the medial thigh to the medial edge of the patella [24].

Taping regions for PT were the same as those for KT, but without any tension, aiming to assess the placebo effect of taping. To prevent any learning effect, the total experiment for each participant lasted for three consecutive weeks, and the washout phase was performed between each taping condition for 1 week. All tapings were conducted by an experienced physiotherapist who did not participate in the recruitment and assessment processes. The anchor length was set at 8 cm (4 cm each for the proximal and distal sites).

### 2.3 Experimental data collection

The experiment was conducted in a biomechanical laboratory. Before the tests, the participants practiced the test movements five times to familiarize themselves with the experimental procedures and performed a 10-minute standardized warm-up. And then 38 reflective markers with a diameter of 14 mm were attached to the participants (left and right anterior superior iliac spine, iliac crest, posterior superior iliac spine, greater trochanter of the femur, lateral femoral condyle, medial femoral condyle, lateral malleolus, medial malleolus, heel, first metatarsal head, fifth metatarsal head, and 4 markers on the side of each thigh and shank). Subsequently, each participant performed three acceptable trials of the 90° pre-planned side-step cut as fast as possible on the affected side [25]. The cutting was considered successful if the participants landed within a force plate and held the landing for more than 2 seconds. If the position of the tested leg moved after the participant landed, it was re-measured until three cuttings were successful, and the average of the three was used for the analysis. A nine-camera infrared high-speed motion capture system (Vicon, T40, 200 Hz) was applied to record the kinematic parameters of the lower limbs during jumping. A three-dimensional force platform (Kistler, 1000 Hz) was used to record the kinetic parameters of the lower limbs during cutting.

### 2.4 Experimental data processing

Kinematic and inverse dynamics analyses were processed using Visual 3D (C-Motion, Inc.). Marker position and force plate data were filtered using a low-pass Butterworth digital filter (12 Hz), which minimizes the artifacts during inverse dynamic analysis in high-impact activities [26, 27]. The initial contact moment was defined as the first time point where the vertical ground reaction force (vGRF) was >50 N [28]. The landing phase was defined as the time interval from post-initial contact to the peak vGRF moment. All kinematic and kinetic variables were extracted for the landing phase. Kinematic variables of interest included peak hip, knee, and ankle angles, and kinetic variables included average hip, knee, and ankle moment, average hip, knee, and ankle power in the sagittal plane, and vGRF. Joint angles were computed as the angles between the proximal and distal segments of the relevant joint. The joint moment was calculated using inverse dynamics based on the kinematic and force plate data. All kinetic variables were normalized by the body weight.

### 2.5 Statistical analysis

Statistical Product and Service Solutions, version 25.0, was used for data processing, using a significance level of 0.05. Descriptive analysis of demographic data included the calculation of frequencies for categorical data and means and standard deviations for continuous data. Normality was confirmed using the Shapiro-Wilk test, and equal variance was confirmed using the Levene test. One-way repeated measures ANOVA was used for kinematic and kinetic variables to determine whether the difference was significant among the taping conditions. Bonferroni post-hoc test was applied to identify the differences.

## 3. Results

### 3.1 Kinematic variables

The knee valgus angle reduced significantly with KT than with NT (95% confidence interval = −1.399 to −0.154; *P* = 0.025), but no significant difference was observed between PT and NT (95% confidence interval = −1.251 to 0.217; *P* = 0.036). No significant differences in the other kinematic variables were observed among the three taping conditions (*P* > 0.05) (**Table 3)**.

**Table 3:** Kinematic variables.

	KT	PT	NT	*F*	*P*
Peak hip flexion angle (°)	24.02±6.23	26.03±7.72	25.7±5.99	3.254	0.056
Peak hip abduction angle (°)	-0.02±3.88	-0.15±4.29	0.17±3.89	0.112	0.886
Peak hip external rotation angle (°)	-7.87±6.65	-8.4±4.94	-7.68±6.22	0.195	0.778
Peak knee flexion angle (°)	26.04±6.08	26.5±6.85	26.32±5.63	0.097	0.905
Peak knee valgus angle (°)	-3.46±2.83	-3.72±3.21	-4.24±3.05	4.278	**0.025** ^#^
Peak knee external rotation angle (°)	1.26±3.64	1.3±5.32	1.69±4.88	0.163	0.836
Peak ankle dorsiflexion angle (°)	6.65±3.42	6.16±2.81	6.6±2.61	0.329	0.705
Peak ankle valgus angle (°)	-14.71±7.01	-14.00±6.19	-14.75±6.33	0.610	0.543
Peak ankle external rotation angle (°)	-14.72±4.13	-14.26±3.37	-14.14±4.65	0.369	0.681

Values are expressed as mean ± SD; KT: Kinesio taping; PT: Placebo taping; NT: No taping

^#^Significantly different between KT and NT.

### 3.2 Kinetic variables

No significant differences were observed for the kinetic variables among the three taping conditions (*P* > 0.05) (**Table 4)**.

**Table 4:** Kinetic variables.

	KT	PT	NT	*F*	*P*
Hip extension moment (Nm/kg)	-0.42±0.2	-0.45±0.17	-0.46±0.15	0.833	0.442
Hip abduction moment (Nm/kg)	-0.57±0.26	-0.56±0.24	-0.52±0.27	0.336	0.700
Hip external rotation moment (Nm/kg)	-0.06±0.08	-0.01±0.08	-0.03±0.08	1.969	0.168
Knee extension moment (Nm/kg)	-0.29±0.15	-0.29±0.17	-0.29±0.14	0.021	0.974
Knee valgus moment (Nm/kg)	0.04±0.21	0.09±0.22	0.11±0.26	1.708	0.198
Knee external rotation moment (Nm/kg)	-0.02±0.06	-0.03±0.06	-0.01±0.04	1.064	0.353
Ankle plantarflexion moment (Nm/kg)	-0.96±0.15	-0.96±0.2	0.15±18	0.123	0.879
Ankle valgus moment (Nm/kg)	0.36±0.13	0.35±0.15	0.37±0.13	0.234	0.776
Ankle external rotation moment (Nm/kg)	0.11±0.05	0.11±0.05	0.13±0.05	2.608	0.097
Hip power (W/Kg)	-0.81±0.45	-0.79±0.37	-0.74±0.36	0.908	0.402
Knee power (W/Kg)	-0.87±0.33	-0.86±0.38	-0.85±0.34	0.073	0.880
Ankle power (W/Kg)	-5.2±1.26	-5.15±1.08	-5.34±1.26	0.504	0.599
vGRF (BW)	1.02±0.11	1.06±0.11	1.05±0.1	1.553	0.226

Values are expressed as mean ± SD; KT: Kinesio taping; PT: Placebo taping; NT: No taping; N, Newton; vGRF, vertical ground reaction force; BW, body weight.

## 4. Discussion

This study aimed to investigate the effect of KT on the lower limb biomechanics during 90° cutting in ACLR athletes. The results showed that although the KT did not improve the kinetic variables, it reduced the knee valgus angle, which could reduce the risk of secondary ACL injury.

### 4.1 Kinematics

Lower knee flexion angles during the landing phase are associated with a higher risk of ACL re-injury [16]. Conventional tapes and braces are thought to improve knee stability; however, they may limit the range of motion of the knee and decrease the knee flexion angle during the landing phase, increasing the risk of ACL injury [29]. The elasticity of KT allows achieving a partial to full range of motion and hence does not limit the range of motion of the joint [30]. Additionally, KT is thought to improve blood circulation, and this physiological change may affect the muscle and myofascial functions and increase the range of motion of the joint [31–33]. However, this study showed no significant difference in the peak knee flexion angle among the three taping conditions during the landing phase. Notably, most of the literature reporting improvements in the knee flexion angle with KT has assessed the effect of KT on knee flexion angle in fixed positions rather than dynamic tasks [31]. Larger knee loads during the landing phase of the dynamic task may mask the positive effects of KT. Botsis et al. [34] noted that the soft tissues of the joints typically stiffen with increased loading during high-load exercises, which may reduce the effect of KT on the range of motion of the joints.

This study demonstrated that KT significantly reduces the knee valgus angle compared to NT and corroborated the findings of a recent study [19]. In the aforementioned study, KT was applied from the tibial tuberosity to the medial and lateral femoral condyles with 75% tension, which might have limited the anterior translation of the tibia and knee valgus. We applied KT to the rectus femoris, medial femoris, and lateral femoris muscles with 50% tension. The tactile stimulation provided by KT might have improved proprioception, increasing the knee stability and decreasing the valgus angle. Simon et al. [35] showed that the application of KT for 72 hours significantly improved ankle proprioception in participants. However, Halseth et al. [36] evaluated the effects of KT on ankle proprioception in healthy participants. The findings indicated that short-term application of KT did not enhance ankle proprioception in the joint position sense test. The difference in the durations of the taping application may have contributed to the conflicting results in the study. The longer the duration of KT application, the higher the chronic stimulation of skin mechanoreceptors [37, 38]. Notably, the reduction in the knee valgus angle was observed even though short-term taping was performed in our study. Long et al. [39] noted that KT enhanced proprioception in participants with poor functional performance but did not show positive effects in those with better physical function. Wei et al. [24] also noted that patients with poor proprioception may be more sensitive to and easier to manage with KT and transfer more proprioceptive information from the joint structures to the nervous system. Hence, patients with poor proprioception experienced more benefits than healthy participants with good proprioception. Considering the reduced stability and poor proprioceptive input in ACLR athletes, KT may be more effective in promoting proprioception.

### 4.2 Kinetics

Joint moments represent the strength of the muscles surrounding the joint, and knee extension moments primarily represent the strength of the quadriceps [40]. The results of this study suggest that the KT did not significantly improve the knee extension moment compared to PT and NT, implying that KT did not improve quadriceps strength. Lins et al. [41] noted that KT application did not improve quadriceps neuromuscular performance during the single-leg jump task. Poon et al. [42] showed that KT did not facilitate muscle performance in generating higher peak torque, yielding a greater total work, or inducing an earlier onset of peak torque. Although most previous studies conducted in this area have reported that KT does not increase quadriceps strength in healthy individuals [43], some studies have shown its positive effects on muscle function. Słupik et al. [44] evaluated the effect of KT on muscle activation in healthy participants and reported a significant increase in muscle activation of the vastus medialis femoris after 24 hours of KT application, and this effect lasted for 48 hours. Several theories have been proposed to explain how KT increases neuromuscular recruitment, including activation of skin receptors, enhancing peripheral afferent signaling, and providing feedback to regulate the central nervous system and peripheral receptors in the joints and muscles [45]. Schleip et al. [46] reported that KT can activate cutaneous mechanoreceptors and induce greater muscle recruitment. According to Mandelbaum et al. [47], these stimuli are critical for neuromuscular control and motor performance. However, Lins et al. [48] used a similar taping protocol as ours to observe the effects of the short-term application of KT on muscle activation. Their results did not show a significant effect of KT on the medial femoral muscle activation. Similarly, our study provided evidence that the knee extension moment did not improve after KT application. This contradicts the proposed mechanism of KT. One possible explanation is that although the tactile input provided by KT can stimulate the cutaneous mechanoreceptors and increase muscle excitability, it is not sufficient to increase muscle strength [49].

Although KT may not provide additional gains in quadriceps strength, Kim et al. [50] noted that KT combined with exercise training significantly improved quadriceps strength in female softball players than that with exercise training alone. This may represent a greater improvement in muscle strength with KT combined with exercise training than with exercise training alone. Therefore, it is recommended to conduct high-quality randomized controlled trials focusing on the effects of KT combined with exercise training on muscle strength. Another aspect to consider is the population in which KT is performed. For individuals with musculoskeletal pain or fatigue, KT could act through its underlying mechanisms. Aghapour et al. [51] showed that the application of KT to the medial femoral muscle with a tension of 75% can reduce pain and improve peak quadriceps torque in patients with patellofemoral pain syndrome. Notably, there was a negative correlation between pain intensity and peak muscle torque. Therefore, the authors attributed the positive effect of KT on peak quadriceps torque to pain reduction. Ahn et al. [52] evaluated the effect of KT on quadriceps strength after fatigue and showed that it could increase the peak quadriceps muscle torque. Fatigue causes muscle pain, which leads to a decrease in peak quadriceps torque [53]. Similarly, we have reason to believe that the positive effect of the KT on peak quadriceps torque after fatigue may be achieved by reducing pain. These studies suggest that in individuals with musculoskeletal pain or fatigue, KT could increase quadriceps torque by reducing pain. However, further studies confirming this are warranted.

### 4.3 Clinical implications

A large knee valgus angle during cutting has been identified as a risk factor for ACL injuries [54]. An important takeaway from this study is that KT significantly reduces the knee valgus angle compared to NT in ACLR athletes during 90° cutting. Notably, KT cannot improve the kinetic variables in athletes after ACLR. This could be because the tactile input provided by KT stimulates the cutaneous mechanoreceptors and increases muscle excitability but is not sufficient to increase muscle strength. Nonetheless, since KT has shown a positive effect in reducing the knee valgus angle, it can help reduce the risk of secondary ACL injury in athletes after ACLR.

### 4.4 Limitations

This study has several limitations. First, the included athletes were not differentiated based on their sports. Second, men and women were not analyzed separately due to the relatively small sample size, despite some evidence of biomechanical differences between the sexes. Additionally, the study may have suffered from a lack of statistical power to detect certain effects in the data due to its relatively small sample size. Finally, considering that most athletes use KT in practice only for a short period, this study only investigated the short-term effects of KT; hence, the results cannot be generalized to long-term effects.

## 5. Conclusion

KT can reduce the knee valgus angle of ACLR athletes during the 90° cutting maneuver, which could reduce the risk of secondary ACL injury.

## Supporting information

S1 Data Raw data. (ZIP)
